# Risk Models for Monitoring Postoperative Complication Rates After Paediatric Cardiac Surgery

**DOI:** 10.1093/ejcts/ezaf317

**Published:** 2025-09-26

**Authors:** Hannah K Mitchell, Ferran Espuny-Pujol, Rodney C Franklin, Gareth Ambler, John Stickley, Julie A Taylor, Carin Van Doorn, Serban Stoica, Victor Tsang, Christina Pagel, Sonya Crowe, Katherine L Brown

**Affiliations:** Infection, Immunity and Inflammation Department, University College London (UCL) Institute of Child Health, London, WC1N 1EH, United Kingdom; Clinical Operational Research Unit, UCL, London, WC1H OBT, United Kingdom; Department of Computer Science, University of Reading, Reading, RG6 6UR, United Kingdom; Pediatric Cardiology Department, Royal Brompton and Harefield NHS Foundation Trust, London, SW3 6NP, United Kingdom; Department of Statistical Science, UCL, London, WC1E 6BT, United Kingdom; Department of Cardiac Surgery, Birmingham Women’s and Children’s NHS Foundation Trust, Birmingham, B4 6NH, United Kingdom; Clinical Operational Research Unit, UCL, London, WC1H OBT, United Kingdom; Department of Pediatric Cardiac Surgery, Leeds General Infirmary, Leeds, LS1 3EX, United Kingdom; Paediatric Cardiac Surgery, Bristol Heart Institute, Bristol, BS2 8BJ, United Kingdom; Great Ormond Street Hospital NHS Biomedical Research Centre London and Institute of Cardiovascular Science UCL, London WC1N 3JH, United Kingdom; Clinical Operational Research Unit, UCL, London, WC1H OBT, United Kingdom; Clinical Operational Research Unit, UCL, London, WC1H OBT, United Kingdom; Great Ormond Street Hospital NHS Biomedical Research Centre London and Institute of Cardiovascular Science UCL, London WC1N 3JH, United Kingdom

**Keywords:** cardiac surgery, outcomes, care quality, complications

## Abstract

**Objectives:**

As postoperative mortality for paediatric cardiac surgery is very low, we aimed to develop methods for monitoring of postoperative complication rates, given their impact upon children’s health and well-being.

**Methods:**

We used national registry data to develop and evaluate a suite of risk adjustment models for the outcomes of 6 defined postoperative complications, designed for use in complication monitoring for quality assurance.

**Results:**

There were 23 423 30-day postoperative episodes in children under the age of 18 years undergoing cardiac surgery between 2015 and 2021 in England and Wales, with 361 (1.5%) deaths <30 days. Two hundred fifty-seven (1.9%) of 13 556 postoperative episodes in infants (<1 year) involved necrotizing enterocolitis; 158 (1.3%) of 12 408 postoperative episodes between 2018 and 2021 involved prolonged pleural effusion; and among the full sample of postoperative episodes, there were 526 (2.2%) acute neurological events, 446 (1.9%) extracorporeal life supports, 740 (3.6%) renal replacement therapies, and 1006 (4.3%) unplanned reinterventions within 30 days of surgery. The risk adjustment models were developed using clinical factors first defined for mortality monitoring. The models for prolonged pleural effusion, extracorporeal life support, and renal replacement performed very well with area under the curve (AUC) statistics >0.85. The performance of the models for necrotizing enterocolitis, acute neurological event, and unplanned reintervention was less good (AUC statistics 0.74-0.79).

**Conclusions:**

Although complications are more complex outcome measures than mortality, national registry data can be used to capture them and to evaluate methods for risk adjustment of these outcomes. These methods may enable future risk-adjusted monitoring of complication metrics for quality assurance.

## Graphical abstract

**Figure:**
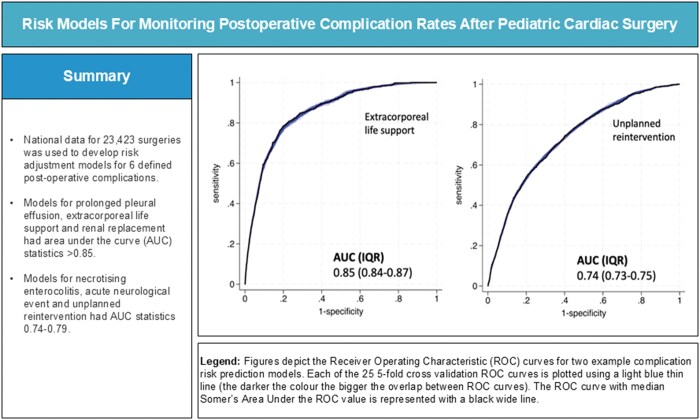

## INTRODUCTION

The 30-day postoperative mortality following paediatric cardiac surgery is <2% in the United Kingdom.^1^ Mortality is monitored using variable life adjusted display charts^2^ reviewed monthly within all centres, and annually for the United Kingdom and Republic of Ireland by the National Congenital Heart Diseases Audit (NCHDA). These analyses, based on risk adjusted analyses of 30-day mortality,^3^^,^^4^ enable the review of recent trends in benchmarked outcomes and support prompt action if any worrisome deviations are observed.

In line with international efforts to supplement postoperative mortality monitoring with additional metrics,^5–7^ the national audit started collecting complication outcomes in 2015, and reporting centre level, unadjusted rates in 2020.^1^ The complication metrics were based on a multicentre prospective research project that selected,^8^ defined,^9^ measured,^10^ and evaluated^11^ 9 complication outcomes. Prospective study noted these selected complications to be important based on demonstrated links to prolonged hospitalization, costs, quality of life,^11^ and toddler neurodevelopment.^12^ Given the feasibility constraints of national audit, mandatory monitoring of complications was restricted to 6 of the 9 metrics, and their definitions refined based on clinician and data manager feedback.^13^

In this study, we aimed to develop risk adjustment methods for each of the 6 complication metrics monitored by the national audit so that future use in local quality assurance and national reporting could take account of case-mix.

## METHODS

### Data sources

We used all records of cardiac surgeries that occurred in public hospitals in England and Wales between 2015 and 2021 for patients aged under 18 years. This data submission is mandatory and subjected to annual external validation. In line with postoperative mortality monitoring,^1^ we created 22 423 “30-day episodes” that started with a cardiac surgery and ended with the patient’s vital status at 30 days. Patients who had only a transcatheter or cardiac support procedure were excluded. Subsequent surgeries for the same patient occurring within a 30-day episode did not count as new 30-day episodes. If a patient had further cardiac surgery more than 30 days later than their index surgery, this initiated a new 30-day episode. All variables were based on the European Paediatric Cardiac Code version of the International Paediatric Cardiac Code.

### Ethical approvals

The study was approved by the North of Scotland National Health Service Research Ethics Committee on February 14, 2020 (20/NS/0022) and the Health Research Authority Confidentiality Advisory Group on July 12, 2020 (20/CAG/0027) which permits the use of routinely collected patient data without consent.

### Complication outcomes

The outcome measures were the following 6 complications, ascertained <30 days of surgery: necrotizing enterocolitis, prolonged pleural effusion, acute neurological event, extracorporeal life support, renal replacement therapy, and unplanned reintervention (comprised any one of; unplanned additional cardiac surgery, interventional catheterizations, permanent pacemaker placements, and diaphragm plication procedures). The detailed definitions (**Appendix S1**) are in the NCHDA data manual.^13^ The time criteria for prolonged pleural effusion changed from 7 to 10 days after surgery in 2018; hence, analysis of this outcome was restricted to post-2018 data. The analysis of necrotizing enterocolitis was restricted to children aged under 1 year at surgery given the age distribution of this complication.

### Candidate risk factors

Candidate risk factors were based on those identified as important in our previous prospective study of postoperative complications.^10^ These were defined by record level codes and followed national audit definitions^3^^,^^4^^,^^14^ (we provide expanded details in **Appendix S2**, and we provide lists of congenital heart diseases (CHDs) and specific operation types in **Tables S1 and S2**).

- Age (in years) and weight (in kg) were included as a continuous term and square root term (the latter to account for the non-linear relationship between age/weight and outcome). Records that had an absolute weight-for-age Z-score of 5 or higher, weights deemed infeasible by a clinician, and missing weights were assigned the average weight-for-age.
- Specific cardiac surgeries (*N*=58) and CHDs (*N*=26) were identified based on clinical codes and national algorithms.
- Additional preoperative risk factors were identified before surgery from record level codes using national definitions: the presence of functionally univentricular heart (FUH),^3^^,^^4^ acquired comorbidity (eg, renal failure),^14^ additional cardiac risk factors (eg, impaired ventricular function),^14^ congenital non-cardiac comorbidity (eg, genetic syndrome),^14^ congenital cardiac risk factors (eg, anomalous coronary artery), Down syndrome, prematurity (<37 weeks birth gestation), increased severity of illness factors present (eg, ventilated),^14^ and level of operation urgency.^13^

### Data processing

To avoid model overfitting, we considered that the number of events in the dataset should be 10 times larger than the number of parameters^15^; therefore, we collapsed the 58 cardiac procedures and 26 CHDs into broader groups. Thus, for each complication outcome, we created 10 specific cardiac operation groups and 8 CHD groups ranked in prevalence order for the relevant complication. For prolonged pleural effusion where there was a lower event number, we limited this to 7 specific cardiac operation groups and 5 CHD groups. All groups were checked by clinical experts to ensure face validity and we report details of these in **Tables S1 and S2**. We marked up 8/348 (2.3%) specific cardiac operation and complication outcome combinations with zero events: these 8 were moved into the mid from the lowest risk band based on clinical opinion that the event number in wider practice is not zero.

### Statistical analysis

We reported missing values and the prevalence of each candidate risk factor by complication outcome, with the relevant Chi square *P*-value for the whole dataset, inclusive of deceased patients. Because death could be a competing event with the occurrence of complications, we calculated the interval in days between the index surgery and death, finding a median of 11-14 days (first quartile 4-5 days) at death in the absence of any of the 6 complication outcomes. The interval from index surgery to complication onset was obtained from a prior, prospective study^10^ as follows (median and interquartile range (IQR) in days): acute neurological event 6 (3–14), prolonged pleural effusion 6 (3–10), extracorporeal life support 1 (0–2), necrotizing enterocolitis 6 (4–18), unplanned reintervention 9 (3–17), and renal replacement therapy 2 (1–2). The short time interval between surgery and onset for extracorporeal life support and renal replacement therapy meant that death was not considered a competing event; hence, in these risk models, all records were included. For the other 4 risk models, we removed the records of patients who died without this complication occurring.

When developing the risk adjustment models, we undertook a complete case analysis. For each outcome, we conducted univariate logistic regression (with standard errors estimated clustering by centre) for all candidate risk factors and selected for inclusion those with *P* < 0.2 in univariate analysis; then multivariate logistic regression backward selection was applied (with *P-*value threshold *P* < 0.2 and standard errors clustering by centre). We thus generated a prospective multiple variable risk model for each outcome. Receiver operating characteristic (ROC) curves, model calibration slope, and calibration-in-the-large and Brier scores were calculated across 25 5-fold cross-validation repeats.

## RESULTS

There were 23 423 30-day postoperative episodes with 361 (1.5%) deaths <30 days. A total of 47 included records involved an imputed weight. Descriptive analyses of complication prevalence involved up to 94 records with a missing value and included all records involving death within 30 days: details for each complication outcome are shown in **Tables 1 and 2**.

**Table 1.** Prevalence of Risk Factors based on Postoperative Complications Ascertained in Selected Samples

	Necrotizing enterocolitis			Prolonged pleural effusion		
Risk factor	Total	No	Yes	Total	No	Yes
All records in sample	13 556	13 299	257	12 408	12 250	158
Weight and age						
Weight (kg) median (IQR)	4.3 (3.2-6.1)	4.3 (3.2-6.1)	3.3 (2.9-4.1)***	7.1 (3.9-15.0)	7.0 (3.9-15.0)	14.8 (6.4-18.6)***
Age (years) median (IQR)	0.2 (0.1-0.5)	0.2 (0.1-0.5)	0.1 (0.0-0.2)***	0.6 (0.2-3.9)	0.6 (0.2-3.9)	3.5 (0.4-5.4)***
Age band			***			***
Neonate (<28 days)	4027 (30%)	3895 (29%)	132 (51%)	2154 (17%)	2133 (17%)	21 (13%)
Infant (28 days-1 year)	9529 (70%)	9404 (71%)	125 (49%)	4987 (40%)	4951 (40%)	36 (23%)
Child (>1 year)				5267 (42%)	5166 (42%)	101 (64%)
Sex						
Male	7643 (56%)	7506 (56%)	137 (53%)	6997 (56%)	6912 (56%)	85 (54%)
Female	5910 (44%)	5790 (44%)	120 (47%)	5409 (44%)	5336 (44%)	73 (46%)
Missing	3 (0%)	3 (0%)	0 (0%)	2 (0%)	2 (0%)	0 (0%)
Clinical factors						
Acquired comorbidity	2047 (15%)	1999 (15%)	48 (19%)	2017 (16%)	1998 (16%)	19 (12%)
Additional cardiac risk	987 (7%)	964 (7%)	23 (9%)	939 (8%)	928 (8%)	11 (7%)
Congenital comorbidity	2669 (20%)	2606 (20%)	63 (25%)*	2671 (22%)	2631 (21%)	40 (25%)
Congenital cardiac risk	179 (1%)	172 (1%)	7 (3%)*	155 (1%)	147 (1%)	8 (5%)***
Down syndrome	1355 (10%)	1341 (10%)	14 (5%)*	1003 (8%)	989 (8%)	14 (9%)
Premature	2485 (18%)	2451 (18%)	34 (13%)*	1680 (14%)	1667 (14%)	13 (8%)*
Severity of illness	2861 (21%)	2790 (21%)	71 (28%)*	1661 (13%)	1647 (13%)	14 (9%)
Functionally univentricular heart	1869 (14%)	1797 (14%)	72 (28%)***	1754 (14%)	1667 (14%)	87 (55%)***
Procedure urgency			***			
Elective	6959 (51%)	6873 (52%)	86 (33%)	8522 (69%)	8402 (69%)	120 (76%)
Urgent	6547 (48%)	6376 (48%)	171 (67%)	3848 (31%)	3810 (31%)	38 (24%)
Missing	50 (0%)	50 (0%)	0 (0%)	38 (0%)	38 (0%)	0 (0%)

* *P* ≤ 0.05 and >0.01,

** *P* ≤ 0.01 and >0.001, and

*** *P* ≤ 0.001.

Abbreviations: kg: kilogram; IQR: interquartile range.

**Table 2.** Prevalence of Risk Factors based on Postoperative Complications Ascertained from the Full Sample

	Acute neurological event			Extracorporeal life support		Renal replacement therapy		Unplanned reintervention	
	Total	No	Yes	No	Yes	No	Yes	No	Yes
All in sample	23 423	22 897	526	22 977	446	22 683	740	22 417	1006
Weight and age									
Weight (kg) median (IQR)	7.0 (3.9-15.1)	7.0 (3.9-15.2)	5.3 (3.4-10.7)***	7.0 (3.9-15.2)	4.1 (3.2-8.7)***	7.1 (4.0-15.4)	3.6 (3.0-6.2)***	7.0 (3.9-15.2)	5.8 (3.5-13.2)***
Age (years) median (IQR)	0.6 (0.2-4.0)	0.6 (0.2-4.0)	0.4 (0.1-1.8)***	0.6 (0.2-4.0)	0.2 (0.0-1.1)***	0.7 (0.2-4.1)	0.0 (0.0-0.5)***	0.6 (0.2-4.0)	0.5 (0.1-3.0)***
Age band			***		***		***		***
Neonate (<28 days)	4027 (17%)	3887 (17%)	140 (27%)	3858 (17%)	169 (38%)	3628 (16%)	399 (54%)	3766 (17%)	261 (26%)
Infant(28 days-1 year)	9529 (41%)	9303 (41%)	226 (43%)	9367 (41%)	162 (36%)	9322 (41%)	207 (28%)	9149 (41%)	380 (38%)
Child(>1 year)	9867 (42%)	9707 (42%)	160 (30%)	9752 (42%)	115 (26%)	9733 (43%)	134 (18%)	9502 (42%)	365 (36%)
Sex									
Male	13 013 (56%)	12 711 (56%)	302 (57%)	12 786 (56%)	227 (51%)	12 588 (55%)	425 (57%)	12 453 (56%)	560 (56%)
Female	F10 405 (44%)	10 181 (44%)	224 (43%)	10 186 (44%)	219 (49%)	10 090 (44%)	315 (43%)	9959 (44%)	446 (44%)
Missing	5 (0%)	5 (0%)	0 (0%)	5 (0%)	0 (0%)	5 (0%)	0 (0%)	5 (0%)	0 (0%)
Clinical factors									
Acquired comorbidity	3559 (15%)	3376 (15%)	183 (35%)***	3481 (15%)	78 (17%)	3405 (15%)	154 (21%)***	3358 (15%)	201 (20%)***
Additional cardiac risk	1747 (7%)	1680 (7%)	67 (13%)***	1664 (7%)	83 (19%)***	1679 (7%)	68 (9%)	1630 (7%)	117 (12%)***
Congenital comorbidity	4875 (21%)	4681 (20%)	194 (37%)***	4754 (21%)	121 (27%)***	4708 (21%)	167 (23%)	4602 (21%)	273 (27%)***
Congenital cardiac risk	370 (2%)	353 (2%)	17 (3%)**	361 (2%)	9 (2%)	352 (2%)	18 (2%)*	331 (1%)	39 (4%)***
Down syndrome	1842 (8%)	1811 (8%)	31 (6%)	1826 (8%)	16 (4%)***	1806 (8%)	36 (5%)*	1761 (8%)	81 (8%)
Premature	3144 (13%)	3063 (13%)	81 (15%)	3084 (13%)	60 (13%)	3074 (14%)	70 (9%)***	3030 (14%)	114 (11%)*
Severity of illness	3281 (14%)	3120 (14%)	161 (31%)***	3111 (14%)	170 (38%)***	3070 (14%)	211 (29%)***	3052 (14%)	229 (23%)***
Functionally univentricular heart	3333 (14%)	3184 (14%)	149 (28%)***	3194 (14%)	139 (31%)***	3121 (14%)	212 (29%)***	3046 (14%)	287 (29%)***
Procedure urgency			***		***		***		***
Elective	15 904 (68%)	15 660 (68%)	244 (46%)	15 755 (69%)	149 (33%)	15 638 (69%)	266 (36%)	15 359 (69%)	545 (54%)
Urgent	7430 (32%)	7148 (31%)	282 (54%)	7133 (31%)	297 (67%)	6956 (31%)	474 (64%)	6970 (31%)	460 (46%)
Missing	89 (0%)	89 (0%)	0 (0%)	89 (0%)	0 (0%)	89 (0%)	0 (0%)	88 (0%)	1 (0%)

* *P* ≤ 0.05 and >0.01,

** *P* ≤ 0.01 and >0.001, and

*** *P* ≤ 0.001.

Abbreviations: kg: kilogram; IQR: interquartile range.

### Complication prevalence

The event number and rate for each complication outcome was necrotizing enterocolitis: 257 (1.9%) of 13 556 postoperative episodes in children <age of 1 year; prolonged pleural effusion: 158 (1.3%) of 12 408 postoperative episodes between 2018 and 2021; and among the full sample of 23 423 postoperative episodes, there were: acute neurological event 526 (2.2%), extracorporeal life support 446 (1.9%), renal replacement therapy 740 (3.6%), and unplanned reintervention 1006 (4.3%).

### Cardiac risk factors and complications

The ranked “risk groups” into which we collapsed 26 CHDs and 58 specific cardiac operations for each complication prevalence and for use in the modelling are reported in **Tables S1 and S2**. For each stated complication, the cardiac operations at highest risk were:

- Necrotizing enterocolitis (cardiac conduit replacement, totally anomalous pulmonary venous connection repair and arterial shunt, biventricular pacemaker placement).
- Prolonged pleural effusion (congenitally corrected transposition repair, Rastelli-REV procedure, Fontan operation).
- Acute neurological event (implantable cardioverter defibrillator operation, totally anomalous pulmonary venous connection repair and arterial shunt, arterial switch, and aortic arch obstruction repair).
- Extracorporeal life support (totally anomalous pulmonary venous connection repair and arterial shunt, heart transplant, truncus and interruption repair).
- Renal replacement therapy (truncus and interruption repair, Senning operation, Norwood stage 1 operation).
- Unplanned reintervention (Rastelli-REV procedure, tricuspid valve replacement, congenitally corrected transposition repair).

Presence of a functionally univentricular circulation was associated with much higher risk of all complications.

### Additional risk factors and complications

Younger age, smaller size, and urgent compared to elective operation were associated with much higher risk of all complications except prolonged pleural effusion (*P* < 0.001 for all). Prolonged pleural effusion was more common in older, larger children and was unrelated to operation urgency.

Strong evidence for an association (*P* < 0.001 for all) was found for:

- Preoperative acquired comorbidity with acute neurological event, renal replacement, and unplanned reintervention.
- Additional cardiac risk factors with acute neurological event, extracorporeal life support, and unplanned reintervention.
- Congenital comorbidity with acute neurological event, extracorporeal life support, and unplanned reintervention.
- Congenital cardiac risk factors with pleural effusion and unplanned reintervention.
- Down syndrome, with extracorporeal life support.
- Preoperative critical illness with acute neurological event, extracorporeal life support, renal replacement, and unplanned reintervention.

With preterm birth, there was much lower risk of renal replacement therapy.

We present additional demographic information in **Tables S3 and S4**.

### Risk models

**Figure 1.**
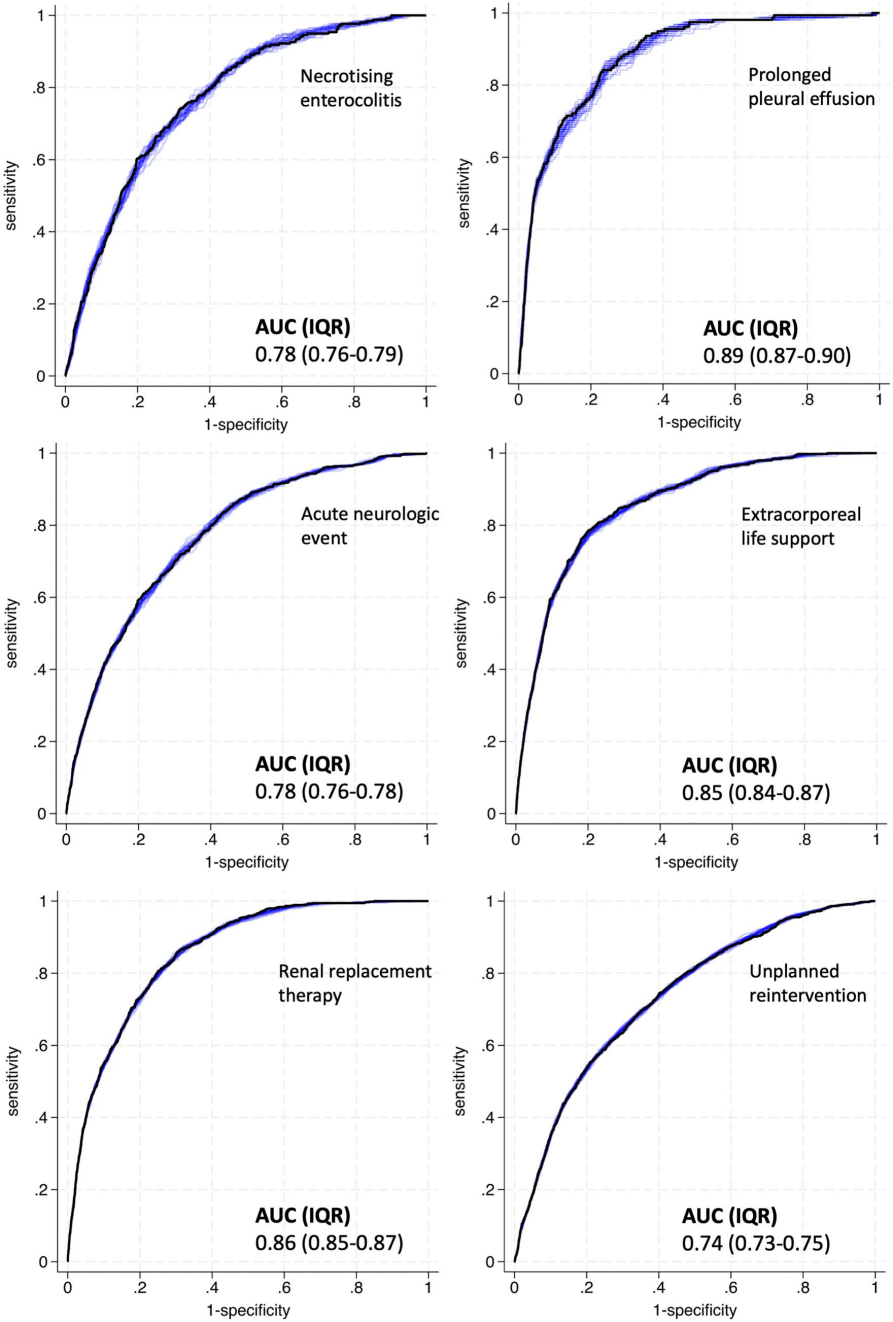
Receiver Operating Characteristic (ROC) Curves for Each of the 6 Complication Risk Prediction Models that Were Developed Using a Sample of National Registry Data Pertaining to 23 423 Paediatric Congenital Cardiac Surgeries. Each of the 25 5-fold cross-validation ROC curves is plotted using a light blue thin line (the darker the colour the bigger the overlap between ROC curves). The ROC curve with median Somer’s area under the ROC value is represented with a black wide line. Abbreviation: AUC: area under the curve.

Up to 94 records that involved a missing value, and records involving death <30 days without the specified complication (necrotizing enterocolitis (*n* = 284), prolonged pleural effusion (*n* = 190), acute neurological event (*n* = 306), and unplanned reintervention (*n* = 301)) were removed. The univariate analysis is presented in **Table S5**, and the multi-variable risk models are presented in **Table 3**, with the total contributing records in first row. The risk models for prolonged pleural effusion, extracorporeal life support, and renal replacement therapy performed very well with area under the curve (AUC) statistic >0.85. The performances of the risk models for necrotizing enterocolitis, acute neurological event and unplanned reintervention were slightly less good (AUC statistics 0.74 to 0.79) (see **Table 3** and **Figure 1** for details).

**Table 3.** Multiple Logistic Regression Models and Model Performance Measures

	Necrotizing enterocolitis	Prolonged pleural effusion	Acute neurological event	Extracorporeal life support	Renal replacement therapy	Unplanned reintervention
Total contributing records	13 219	12 178	23 023	23 329	23 329	23 028
Risk factor						
Sex girl v boy		1.18 (1.05, 1.33)**		1.36 (1.12, 1.66)**		
Age in years					1.37 (1.18, 1.60)***	1.04 (0.99, 1.09)
Age (SQRT)	2.72 (1.04, 7.11)*	1.75 (1.25, 2.44)***			0.45 (0.25, 0.83)*	
Weight in kg		0.97 (0.96, 0.99)**		1.02 (0.99, 1.05)		0.99 (0.97, 1.00)
Weight (SQRT)	0.13 (0.07, 0.24)***			0.76 (0.59, 1.00)*	0.64 (0.41, 1.02)	
Urgent v elective types			1.58 (1.32, 1.90)***	1.92 (1.35, 2.74)***	1.40 (1.15, 1.70)***	1.44 (1.32, 1.56)***
Acquired comorbidity	1.31 (0.99, 1.71)		2.42 (1.50, 3.91)***		1.57 (1.11, 2.21)*	1.26 (0.98, 1.63)
Additional cardiac risk				1.90 (1.39, 2.59)***		1.25 (1.05, 1.49)*
Congenital comorbidity	1.27 (0.93, 1.72)	1.33 (1.12, 1.59)**	2.10 (1.79, 2.47)***	1.44 (1.03, 2.00)*		1.28 (1.04, 1.57)*
Congenital cardiac risk						1.77 (0.99, 3.18)
Down syndrome						
Prematurity	0.66 (0.46, 0.94)*					
Severity of illness			1.68 (1.25, 2.26)***	2.15 (1.30, 3.55)**	1.33 (1.04, 1.71)*	1.51 (1.08, 2.11)*
Functionally univentricular heart	1.88 (0.86, 4.10)	2.39 (1.44, 3.98)***	1.34 (0.93, 1.93)	1.34 (0.97, 1.86)		
Procedure prevalence groups						
(2 v 1)	1.90 (0.76, 4.74)***	5.47 (1.34, 22.32)***	1.74 (0.89, 3.39)***	5.08 (1.68, 15.34)***	4.75 (2.09, 10.80)***	1.28 (0.49, 3.38)***
(3 v 1)	1.61 (0.46, 5.67)***	11.57 (4.59, 29.18)***	2.27 (1.35, 3.80)***	6.78 (3.65, 12.61)***	7.29 (2.73, 19.44)***	1.53 (0.56, 4.20)***
(4 v 1)	7.46 (3.20, 17.37) ***	15.08 (4.85, 46.82)***	2.68 (0.99, 7.24)***	9.94 (5.64, 17.52)***	15.38 (5.87, 40.29)***	1.29 (0.47, 3.56)***
(5 v 1)	3.85 (1.36, 10.94)***	25.16 (8.65, 73.18)***	3.59 (2.11, 6.11)***	11.36 (5.15, 25.01)***	25.60 (12.19, 53.77)***	2.57 (1.08, 6.09)***
(6 v 1)	4.23 (1.76, 10.15)***	35.29 (12.32, 101.13)***	4.44 (2.33, 8.44)***	11.70 (6.55, 20.90)***	40.53 (13.26, 123.91)***	1.58 (0.66, 3.80)***
(7 v 1)	5.71 (2.84, 11.48)***	50.06 (21.41, 117.01)***	4.41 (2.19, 8.91)***	9.15 (4.70, 17.81)***	41.72 (21.99, 79.13)***	2.62 (1.05, 6.53)***
(8 v 1)	8.24 (4.14, 16.37)***	80.63 (25.38, 256.22)***	5.38 (2.13, 13.57)***	21.36 (9.91, 46.02)***	75.56 (42.51, 134.31)***	2.82 (1.19, 6.66)***
(9 v 1)	7.98 (3.30, 19.29)***		6.46 (3.99, 10.46)***	23.20 (11.12, 48.41)***	123.30 (49.54, 306.90)***	3.31 (1.24, 8.79)***
(10 v 1)	15.79 (7.06, 35.33)***		8.53 (4.15, 17.54) ***	40.66 (17.79, 92.94)***	90.76 (37.70, 218.51)***	6.19 (2.39, 16.08)***
CHD Prevalence Groups						
(2 v 1)	5.71 (1.99, 16.37)***	3.07 (1.22, 7.71)***	2.75 (1.05, 7.22)***	1.65 (0.46, 6.01)***	1.37 (0.60, 3.14)***	3.52 (1.25, 9.89)***
(3 v 1)	5.56 (2.31, 13.36)***	4.38 (1.56, 12.30)***	4.29 (1.66, 11.04) ***	2.79 (0.86, 9.08)***	1.87 (0.68, 5.17)***	5.65 (1.97, 16.18)***
(4 v 1)	7.38 (1.86, 29.30)***	3.88 (0.99, 15.12)***	5.66 (2.46, 12.99)***	3.86 (1.11, 13.42)***	2.18 (0.93, 5.08)***	6.38 (2.03, 20.03)***
(5 v 1)	5.00 (1.68, 14.86)***	5.97 (2.06, 17.29)***	5.08 (1.68, 15.32) ***	3.05 (0.94, 9.87)***	1.93 (0.82, 4.56)***	7.35 (2.98, 18.13)***
(6 v 1)	6.55 (2.30, 18.62)***	2.83 (1.27, 6.34)***	6.44 (2.66, 15.60)***	5.22 (1.62, 16.84)***	2.70 (1.23, 5.92)***	11.32 (4.21, 30.45)***
(7 v 1)	12.16 (4.11, 35.97)***		8.20 (3.89, 17.29)***	5.70 (2.13, 15.25)***	1.66 (0.89, 3.12)***	13.02 (5.07, 33.43)***
(8 v 1)	6.11 (2.86, 13.04)***		6.34 (2.43, 16.50)***	4.42 (1.47, 13.28)***	2.53 (1.14, 5.64)***	13.78 (5.12, 37.08)***
In-sample validation						
Area under ROC curve	0.79	0.90	0.78	0.86	0.86	0.74
Number of procedures	2654	2443	4607	4632	4666	4608
Cross validation						
Calibration slope median (IQR)	0.90 (0.79, 0.99)	0.91 (0.85, 1.06)	0.95 (0.89, 1.00)	0.94 (0.90, 1.02)	0.97 (0.91, 1.03)	0.95 (0.89, 1.02)
Calibration-in-the-large median (IQR)	−0.010 (−0.12, 0.12)	0.015 (−0.15, 0.16)	0.004 (−0.09, 0.07)	−0.009 (−0.10, 0.10)	0.012 (−0.07, 0.06)	−0.006 (−0.05, 0.05)
Area under ROC curve median (IQR)	0.78 (0.76, 0.79)	0.89 (0.87, 0.90)	0.78 (0.76, 0.78)	0.85 (0.84, 0.87)	0.86 (0.85, 0.87)	0.74 (0.73, 0.75)
Brier score median (IQR)	0.018 (0.02, 0.02)	0.012 (0.01, 0.01)	0.022 (0.02, 0.02)	0.018 (0.02, 0.02)	0.028 (0.03, 0.03)	0.040 (0.04, 0.04)

For each complication outcome, we present the adjusted odds ratio with 95% confidence interval and *P*-values as

* *P* ≤ 0.05 and >0.01,

** *P* ≤ 0.01 and >0.001, and

*** *P* ≤ 0.001.

Calibration-in-the-large is assessed by comparing the average predicted risk with the observed event rate: a perfectly calibrated model should have a value of 0.

Brier score is the mean squared difference between predicted probabilities and actual outcomes, with 0 meaning perfect prediction.

Abbreviations: SQRT: square root, IQR: interquartile range, CHD: congenital heart disease; ROC: receiver operating characteristic.

## DISCUSSION

We aimed to develop a suite of 6 risk adjustment models for routine quality assurance processes in paediatric cardiac surgery when assessing complication rates. Hence, to make interpretation as clear as possible for users, we used a similar data management and statistical approach across all 6 of the models. Although complication outcomes are more complex metrics than 30-day mortality, it was feasible to develop risk models for case mix adjustment that could be taken forwards by the national audit. The outcomes that could be clinically ascertained most consistently had the best performing risk models (prolonged pleural effusion, extracorporeal life support, and renal replacement therapy). Ascertainment of necrotizing enterocolitis, acute neurological event, and unplanned reintervention entail consideration of several complex clinical parameters, potentially contributing to their weaker model performances.

### Context

The Society of Thoracic Surgery Congenital Heart Surgery Database monitors a range of complication metrics closely matching those selected by NCHDA (unplanned reinterventions inclusive of diaphragm plication and permanent pacemakers, renal replacement therapy and new neurological deficits) and has stressed the importance of these metrics in quality assurance.^6^^,^^7^ The Paediatric Cardiac Critical Care Consortium (PC4) successfully demonstrated that reporting and review of complication metrics (which include cardiac arrest, mechanical circulatory support, unplanned cardiac reintervention, neurologic complications, chylothorax) can lead to improved outcomes of mortality and length of stay.^5^ The success of PC4 and our alignment with some PC4 metrics supports the hypothesis that our study has potential to benefit future patients in England and Wales. The near real-time monitoring of risk adjusted 30-day mortality rates of paediatric cardiac surgery has been helpful to clinical teams in the United Kingdom, and we hope to test a similar process for complication monitoring.

### Strengths and limitations

We present unique descriptive information about the rates and risk factors for selected important complications linked to paediatric cardiac surgery, including complication prevalences with specific operations. However, we are aware that complication definitions are more variable and open to interpretation than 30-day mortality. We note that the national complication definitions have been subject to refinements to improve clarity: this might mean outcome ascertainment was imperfect. The selected complications used for national audit in the United Kingdom and Republic of Ireland do not capture every possible metric: for example, complications considered important by clinicians that were not included were tracheostomy because this is very rare in our population; surgical site infection because this was not reliably captured; and postoperative cardiac arrest, which was recently added as a national metric.

### Conclusions and next steps

These methods may enable future risk adjusted monitoring of complication metrics for quality assurance. If the risk models are used for risk adjusted routine monitoring of these outcomes, then submitted data quality is likely to improve.

## Supplementary Material

ezaf317_Supplementary_Data

## Data Availability

The study data are held and can only be analysed based on a current and valid data sharing agreement with the National Congenital Heart Diseases Audit and National Health Service Digital.
